# Ultrasound-targeted microbubble destruction: a prospective strategy for treating cardiovascular disease

**DOI:** 10.3389/fcvm.2025.1634059

**Published:** 2025-09-01

**Authors:** Shuting Yang, Rong Zhang, Huali Zhang, Yongping Lu

**Affiliations:** Department of Ultrasound, The Affiliated Hospital of Yunnan University, The Second People’s Hospital of Yunnan Province, Kunming, China

**Keywords:** cardiovascular disease, ultrasound, microbubble, ultrasound targeted microbubble destruction (UTMD), blood-brain barrier (BBB), atherosclerosis

## Abstract

In China, the healthcare burden of cardiovascular diseases (CVDs) will continue to rise due to the pressure of the aging population, which has posed higher demands for CVDs prevention and treatment. Ultrasound-targeted microbubble destruction (UTMD) is an ultrasound-triggered drug delivery technique based on microbubbles. This technique utilizes the principles of cavitation and sonoporation to enhance the delivery of genes or drugs to target tissue. This review article will provide an overview of studies using UTMD to treat CVDs over the last decade. In light of these studies, we underscore the potential therapeutic targets and delineate the practical substances that can be loaded onto microbubbles. Additionally, a discussion is provided regarding the limitations and prospects of this field.

## 1 Introduction

Cardiovascular diseases (CVDs) remains the predominant cause of mortality and premature death in China, accounting for 48% and 45.86% of total deaths in rural and urban populations respectively (1, 2). Atherosclerosis, the most prevalent form of CVDs, manifests as lipid accumulation and chronic inflammation in large arteries, leading to critical complications including ischemic heart disease and cerebrovascular accidents (3). Over the past three decades, China has faced escalating challenges in CVDs management due to the dual pressures of rising atherosclerotic cases and demographic aging, which necessitates innovative approaches for prevention and therapeutic intervention (4).

**Figure 1:**
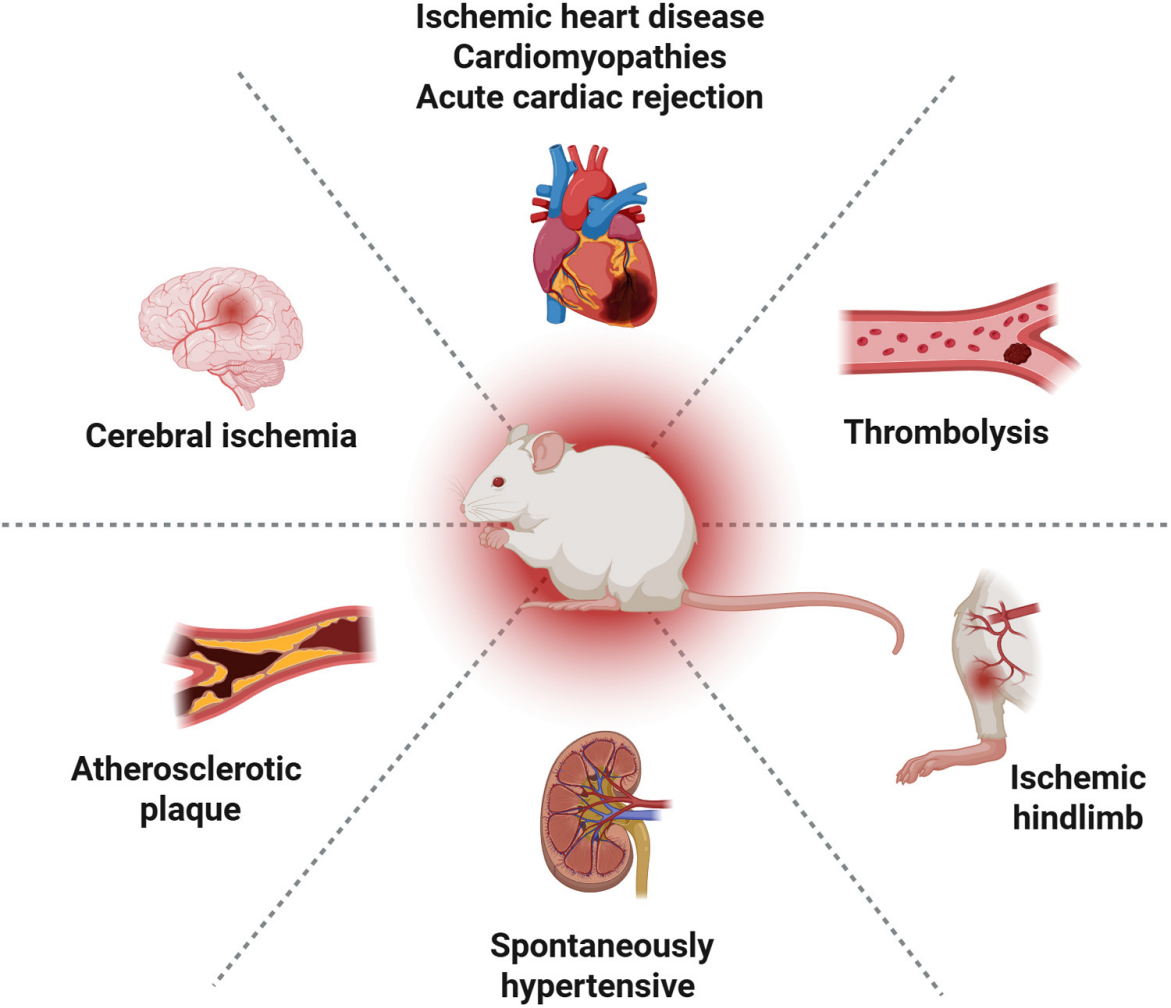
Preclinical evaluation of UTMD in cardiovascular therapy. Created in BioRender. YANG, S. (2025) https://BioRender.com/pdf8y59, licensed under Academic license.

Ultrasound has a long history as a diagnostic imaging technique. Additionally, it has the capacity to modulate the spatial and temporal release of medication. Due to its biocompatibility and low attenuation in tissue, ultrasound demonstrates the potential for remote activation, which has driven the development of smart medicine delivery systems. In recent years, this field has expanded to therapeutic applications, such as ultrasound-targeted microbubble destruction (UTMD) (5, 6). The non-invasive and target-specific nature of UTMD renders it a promising drug and gene delivery strategy (7). Recent studies suggest a promising future for UTMD-based therapies in the management of CVDs (8–10), indicating a potential for advancement in the field of precision medicine (Figure 1).

A systematic literature search was conducted in PubMed using the key term “ultrasound-targeted microbubble disruption” to evaluate the therapeutic advancements of UTMD in CVDs over the past decade. Particular emphasis was placed on innovative approaches for microbubble functionalization. Furthermore, a critical examination of current technological limitations and translational challenges is undertaken to inform future research directions.

## 2 Overview of UTMD

Ultrasound serves as a critical diagnostic modality providing non-invasive real-time imaging capabilities in clinical practice. Microbubbles, when employed as contrast agents, significantly enhance the assessment of tissue perfusion, hemodynamic parameters, and pathological features such as lesions or vascular abnormalities — capabilities that establish them as indispensable tools for the diagnosis and clinical evaluation of cardiovascular diseases (11).

In 1998, Dr. Price and colleagues advanced the notion of UTMD in a publication in the “*Circulation*” journal (12). Over recent decades, UTMD has evolved into a prominent therapeutic strategy. Ultrasound-mediated techniques have garnered significant attention for their tripartite capability in precisely controlling drug activation, real-time monitoring, and spatiotemporally regulated release. Functioning as acoustic energy transducers, microbubbles serve as critical vectors for ultrasound-triggered delivery of both chemical agents and engineered nanoparticles. Several methods are employed to prepare microbubbles, such as atomization and reconstitution, cross-linking polymerization, and emulsion solvent evaporation (13). Gas-filled microbubbles (MBs) with diameters of 1–8 μm are conventionally employed as intravascular ultrasound contrast agents. The gaseous core provides the necessary echogenicity, while the surrounding shell prevents rapid dissolution of the core, thereby ensuring MB stability. These shells may consist of albumin, surfactants, phospholipids, proteins, mesoporous silica, or biocompatible and biodegradable polymers (14, 15). The utilization of insoluble, high-density inert gases—such as perfluorocarbons and sulfur hexafluoride—as core materials significantly enhances microbubble stability. Due to their low blood solubility, these gases prolong microbubble circulation within the vascular system. For instance, air-filled contrast agents (e.g., Albunex and Levovist) exhibit circulation times of approximately 5 min. In contrast, microbubbles containing fluorinated gases (e.g., SonoVue and Optison) demonstrate circulation times exceeding 10 min (16).

**Figure 2:**
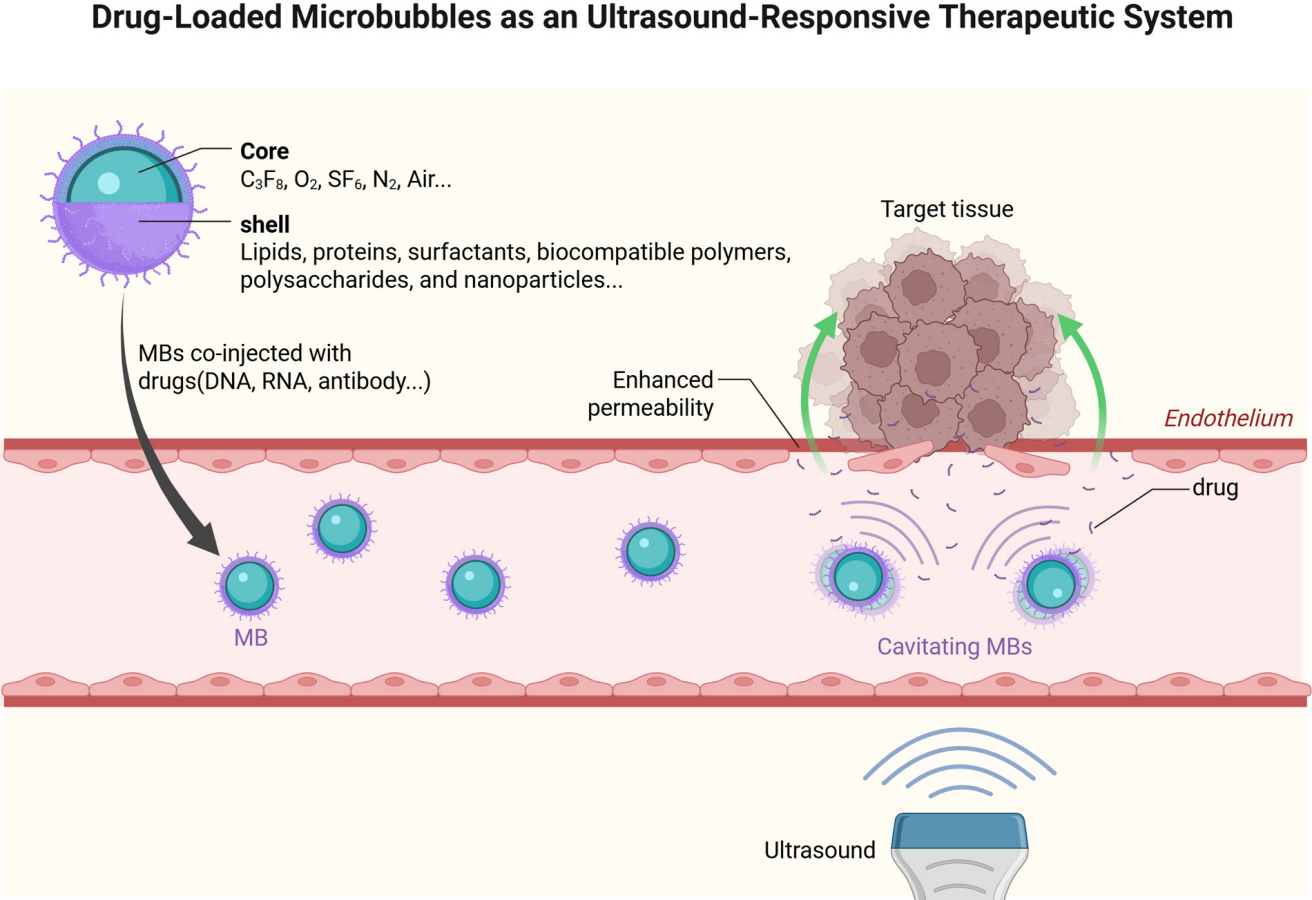
Drug-Loaded microbubbles as an ultrasound-responsive therapeutic system. Created in BioRender. YANG, S. (2025) https://BioRender.com/18kn4mw, licensed under Academic license.

A significant disparity in their capabilities of drug delivery was observed due to substantial discrepancies in microbubble shell composition, gaseous composition, and size distribution. In addition, through surface functionalization or core-loading strategies, these microcarriers can encapsulate diverse therapeutic payloads including small-molecule drugs, nucleic acids, and immunotherapeutic antigens. Following intravenous administration, the acoustically driven inertial cavitation of microbubbles at target sites generates localized shear stresses and microstreaming effects, enabling site-specific payload release with enhanced bioavailability (Figure 2) (15).

The mechanical effects induced by ultrasonic fields, including acoustic radiation force, microstreaming, shear stress, and related phenomena, stem from the momentum transfer associated with sound waves. The occurrence of more substantial mechanical effects is plausible when the MBs interact with ultrasonic beams (17). In the context of low acoustic pressures, these MBs undergo symmetrically periodic expansion and compression, oscillating synchronously with the incident ultrasonic wave. The process in question is referred to as “stable cavitation.” During this process, the MBs undergo an expansion phase in which they extend to the point of coming into proximity with the blood vessel wall. This expansion causes the adjacent endothelium to separate from its neighbors. During the subsequent compression phase, the MBs undergo a process of shrinkage, causing invaginations in the endothelial cells that line the vessel. This process disrupt the tight junctions between the endothelial cells, resulting in a disruption of the tissue's structural integrity (18, 19). In the presence of elevated acoustic pressures, MBs undergo a violent collapse during the compression phase. This process is referred to as inertial cavitation. The propensity of microbubbles to undergo sustained stable cavitation or inertial cavitation depends on multiple factors, chief among which is the mechanical index (MI)—defined as the peak negative acoustic pressure divided by the square root of the ultrasound frequency. The collapse of MBs has been demonstrated to generate shock waves of increased strength, as well as microstreaming, micro-jetting, and tangential stresses. These phenomena have been observed to result in the perforation of cellular membranes. Impulse forces exerted on the endothelial cell membrane can directly result in the formation of transient, nonselective, and repairable pores, a phenomenon referred to as the sonoporation effect (15, 20).

Experimental evidence confirms that microbubble-mediated vascular permeability enhancement—driven by ultrasound-induced shear stress (e.g., 1 MHz, MI = 0.8)—is typically transient and occurs without vascular rupture when using pulsed ultrasound (0.1–10 ms pulses) with low duty cycles, restoring endothelial barrier function within minutes to hours while triggering Ca^2+^ signaling. However, excessive acoustic energy [e.g., high mechanical index (MI >1.3), prolonged burst length, or extended exposure] can induce violent microbubble collapse, causing localized endothelial damage through large-amplitude oscillations, endothelial cell death due to delayed pore resealing, and—under extreme conditions (MI 1.3–2.0)—vascular rupture and hemorrhage (21). Critical damage thresholds depend on both acoustic parameters (pulse scheme, microbubble properties/concentration) and biological factors (calcium-dependent repair mechanisms, membrane composition), as well as tissue-specific vessel characteristics (22).

Microbubble suspensions exhibit excellent biocompatibility as delivery platforms, demonstrating minimal systemic toxicity and negligible immunogenicity (23). This favorable safety profile stems from the fate of their components: Perfluorocarbon (PFC) gases (e.g., perfluoropropane, sulfur hexafluoride) are chemically inert, non-metabolized, and primarily eliminated via exhalation through the lungs within minutes to hours after administration. Meanwhile, the encapsulating shell materials (lipids, proteins, polymers) undergo biodegradation and renal clearance. Supporting this safety, Qin et al.'s cardiomyopathy study using repeated ultrasound-mediated delivery (three sessions at 1-day intervals) of Sirt3 plasmid via cationic microbubbles suppressed hypertrophic phenotypes—including cardiac enlargement, fibrosis, and apoptosis—at both 7 days and 2 months post-treatment. Critically, multi-organ assessment (kidneys, liver, lungs) confirmed no treatment-related damage, demonstrating high safety with minimal off-target effects (24).

Clinically administered via peripheral intravenous injection, these contrast agents enable non-invasive ultrasound-targeted microbubble destruction (UTMD) procedures—a key translational advantage for therapeutic applications (25). Microbubbles crucially protect encapsulated bioactive molecules from enzymatic degradation and immune clearance during circulation. Subsequent ultrasound-triggered inertial cavitation then enables site-specific payload release. This mechanism enhances therapeutic precision while minimizing off-target effects. Furthermore, microbubbles with functionalized surfaces achieve molecular-level targeting, significantly improving spatiotemporal control in diagnostics and therapeutics. Preclinical evidence confirms that molecularly targeted microbubbles substantially enhance gene delivery efficiency compared to non-targeted agents. For instance, in cardiovascular models, Xie et al. demonstrated the feasibility of endothelial-targeted (P-selectin/ICAM-1) gene-carrying microbubbles, achieving a 5-fold higher transfection efficiency than non-targeted controls at MI 0.6 in murine hindlimb ischemia (26). Similarly, Zhou et al. used ICAM-1-targeted microbubbles to deliver Ang1 in rabbit myocardial infarction models, yielding approximately 3-fold greater delivery efficiency vs. non-targeted bubbles using a Philips iE33 system (1.7 MHz, MI 1.3) (27).

## 3 Potential application of UTMD in cardiovascular diseases

### 3.1 Ischemic heart disease

#### 3.1.1 Myocardial infarction (MI)

As a common cardiovascular disease, myocardial infarction (MI) is characterized by the irreversible necrosis of myocardium due to oxygen deprivation. MI typically progresses to impaired diastolic function, myocardial fibrosis, malignant arrhythmia, weakened ventricular contraction, heart failure (HF), and even sudden death (28). Although revascularization is performed via stenting or bypassing of the infarcted artery, ventricular dysfunction remains inescapable after an extensive MI (29). Ultrasound-targeted microbubble destruction UTMD can facilitate drug, gene, and cell delivery into the infarcted heart. Current research demonstrates feasible targeting ligands, such as P-selectin (30) and ICAM-1 (31), which are expressed in endothelial cells, as well as glycoprotein IIb/IIIa receptors on activated platelets (14). In rats, UTMD-mediated local transfection of VEGF (30), miR-150-5p (32), PHD2 (33), Gal-3 (34), and SERCA2a-Cx43 (35, 36) has been used to protect the heart from complications following acute MI. Despite ongoing debate over the efficacy of cardiac stem cell therapy, a recent study suggests that the underlying biological mechanism involves an acute sterile immune response to improve heart function (37). For example, UTMD-delivered PHD2 shRNA-modified BMSCs were shown to enhance grafted cell homing, activity, and myocardial angiogenesis in infarcted hearts (38). Additionally, studies on Ang-1 delivery in rabbits (31), and microRNA-21 delivery in pigs (39) further demonstrate the therapeutic potential of UTMD in treating post-MI cardiac injury.

#### 3.1.2 Ischemia/reperfusion injury (I/R)

Early and successful revascularization can significantly improve clinical outcomes in patients with acute MI. However, reperfusion may paradoxically exacerbate myocardial damage, a phenomenon termed ischemia-reperfusion (I/R) injury. Ischemia initiates inflammatory cascades, and the subsequent restoration of blood flow further activates additional inflammatory pathways, thereby amplifying this paradoxical injury (40). To enhance tissue-specific targeting, a feasible strategy involves conjugating thiolated MMP2 antibodies to cationic microbubbles (41). Experimental studies have demonstrated direct cardiac delivery of therapeutic agents to mitigate I/R injury: in mice, Atagomir (42), TRAF3IP2 (43), GDF11 (44), and in rats, Timp3 (41), S100A6 (45), and hydrogen sulfide (46) were successfully utilized to protect myocardial tissue.

### 3.2 Cardiomyopathies

#### 3.2.1 Diabetic cardiomyopathy (DCM)

Diabetes mellitus and its complications represent a significant global health burden, affecting populations across both developed and developing nations. Cardiovascular pathologies constitute the predominant cause of mortality in diabetic populations, with diabetic cardiomyopathy (DCM) emerging as a distinct myocardial disorder independent of hypertension or coronary artery disease (47). This metabolic cardiomyopathy, driven by chronic hyperglycemia, insulin resistance, and compensatory hyperinsulinemia, manifests through characteristic pathological progression: mitochondrial oxidative stress initiates cardiomyocyte apoptosis, followed by extracellular matrix remodeling (myocardial fibrosis), compensatory hypertrophy, impaired ventricular relaxation (diastolic dysfunction), and ultimately progresses to impaired cardiac contraction (systolic failure) (48). Despite significant advancements in glucose-lowering therapy for diabetes in recent years, conventional medications have proven ineffective in halting the progression of diabetic cardiomyopathy (49).

Animal experiments indicate that acidic fibroblast growth factor (aFGF) and basic fibroblast growth factor (bFGF) are involved in regulating cardiac angiogenesis and repair, suggesting their potential as therapeutic agents for the treatment of DCM (50). However, conventional intracardiac administration poses significant limitations due to invasiveness and procedural risks, hindering clinical translation. This therapeutic impasse has driven innovation in targeted delivery systems, with UTMD demonstrating remarkable efficacy in preclinical models. Mechanistic studies reveal that UTMD-mediated aFGF delivery attenuates ventricular remodeling through activation of PI3 K/Akt signaling pathways (51), while bFGF administration via UTMD enhances angiogenesis via VEGF upregulation and improves cardiac function parameters in DCM models (52, 53). Moreover, PEGylated nanoliposomes could serve as suitable carriers to enhance the stability of non-mitogenic aFGF (NM-aFGF) during storage and systemic circulation (54).

Emerging gene therapy approaches utilizing UTMD demonstrate particular promise. Targeted delivery of S-adenosylhomocysteine hydrolase (SAHH) via UTMD technology has shown capacity to restore ventricular function in DCM. The cardioprotective effects appear mediated through activation of the energy-sensing AMP-activated protein kinase (AMPK)/forkhead box O3 (FOXO3)/sirtuin 3 (SIRT3) axis, a critical pathway modulating mitochondrial biogenesis and oxidative stress responses (55). These findings demonstrate UTMD's utility as a precision cardiac therapeutic platform, enabling site-specific treatment delivery with minimized systemic toxicity.

#### 3.2.2 Cardiotoxic damage

Doxorubicin (DOX), also known as Adriamycin (ADM), is a potent chemotherapeutic agent widely used in the treatment of multiple malignancies. However, its clinical utility is significantly limited by dose-dependent cardiotoxicity, often leading to cardiomyopathy and posing critical therapeutic challenges (56). Resolving this therapeutic dilemma necessitates novel cardioprotective approaches. Recent preclinical studies utilizing doxorubicin-induced cardiomyopathy rodent models have validated UTMD-facilitated precision delivery of multiple therapeutics - including GLP-1 (57), MaFGF (58), ANGPTL8 (59), survivin (60), and aFGF (61) - demonstrating capacity to ameliorate or reverse the established ADM cardiomyopathy. Exosome-mediated nucleic acid delivery has shown therapeutic promise, but its clinical application remains constrained by poor heart-targeting efficiency. The UTMD platform addresses this challenge by enabling precise cardiac targeting, having successfully delivered siHomox1 (62) and miR-21 (63), with demonstrated efficacy in mitigating doxorubicin-induced myocardial damage and functional decline.

Sepsis has been identified as the foremost cause of mortality within intensive care units, accounting for approximately one-fourth of all cases. Concomitant heart dysfunction has been demonstrated to increase the risk of mortality in patients with severe sepsis. Despite the evident decline in cardiac performance observed in patients with sepsis, there remains a paucity of consensus or guidelines regarding the management of sepsis-induced cardiomyopathy (64). Notably, in septic cardiomyopathy models, the UTMD-mediated cardiac delivery of ROR*α* significantly enhanced melatonin's cardioprotective effects in sepsis. Given the favorable biocompatibility, safety profile, and delivery efficiency of UTMD technology, the combined therapeutic approach of melatonin with RORα/cationic microbubbles may represent a promising strategy for managing sepsis-induced cardiomyopathy (65).

#### 3.2.3 Cardiac hypertrophy

Pathological cardiac hypertrophy develops through multiple etiological pathways, encompassing ischemic injury (myocardial infarction), structural valvular abnormalities (aortic stenosis, mitral/aortic regurgitation), metabolic dysregulation in storage disorders, chronic pressure overload (hypertension), and heritable mutations affecting sarcomeric protein genes. Distinct from the compensatory mechanisms of physiological hypertrophy, this maladaptive process evolves through aberrant molecular signaling cascades (66), ultimately culminating in heart failure decompensation, fatal arrhythmogenesis, and sudden cardiac death. Emerging therapeutic interventions utilizing UTMD technology show significant preclinical efficacy. In porcine models of myocardial hypertrophy, UTMD-facilitated Sirt3 gene delivery demonstrates therapeutic efficacy by enhancing sustained cardiac functional recovery while attenuating pathological myocardial remodeling (24). Furthermore, UTMD-mediated antimiR-23a delivery achieves targeted myocardial inhibition of pathological hypertrophy and preservation of left ventricular systolic performance at a dosage 200-fold lower than systemic administration (67).

### 3.3 Acute cardiac rejection

In the absence of contraindications, heart transplantation (HT) remains the standard therapeutic intervention for end-stage heart failure. However, acute rejection (AR) persists as a prevalent complication during the first post-transplant year and is independently associated with accelerated cardiac allograft vasculopathy and irreversible graft dysfunction, significantly impacting long-term outcomes (68). Current AR management relies on high-dose systemic immunosuppression, including calcineurin inhibitors, corticosteroids, and polyclonal antibody therapies. These regimens carry substantial toxicity profiles, manifesting as nephrotoxicity, metabolic derangements (hypertension, hyperlipidemia, glucose metabolism dysregulation), neurocognitive impairment, opportunistic infections, and elevated malignancy risk—collectively diminishing quality of life and threatening post-transplant survival (69). UTMD presents a new, low side - effect approach for AR - targeted therapy. Compared to direct sirolimus administration, ultrasound-targeted microbubbles carrying sirolimus can achieve a local drug concentration 15 times higher (70). In gene therapy, UTMD has been combined with efficient FK506 (71), galectin-7-siRNA (72), and Antagomir-155 (73) delivery methods to treat AR.

### 3.4 Atherosclerotic plaque

Atherosclerosis poses a significant threat to cardiovascular health, with plaque rupture being a primary trigger for acute complications. These rupture-prone plaques are typically characterized by lipid-rich cores, frequent intraplaque hemorrhage, and thin collagen caps that are infiltrated with inflammatory cells (74). This pathological process is the leading cause of cardiovascular disease morbidity and mortality. However, achieving effective localized therapeutic intervention for atherosclerotic plaque lesions remains a substantial clinical challenge. Current treatment strategies are often hampered by inadequate target-specific, which compromise both treatment precision and biological efficacy (75).

Recent studies have demonstrated the potential of various therapeutic agents in stabilizing vulnerable plaques and even inhibiting atherosclerotic plaque progression. For instance, in ApoE^−/−^ mice, the application of rapamycin (76), microRNA-145 (77), Endostar (78), and Nox2 (75) has shown promise in stabilizing plaques. Similarly, in rabbits, the use of GSK-3β (79), IL-8 monoclonal antibody (80), TGF-β1, and TIMP1 (81) delivered via UTMD, has also yielded favorable outcomes. Collectively, these findings underscore the potential of UTMD as a practical and promising technique for the efficient and safe management of atherosclerosis.

### 3.5 Thrombolysis

Thrombosis remains a major global cause of morbidity and mortality by triggering vascular occlusion and subsequent cardiovascular events like acute myocardial infarction, ischemic stroke, and pulmonary embolism (82). To address this, endovascular sonothrombolysis—a technique using ultrasound-enhanced clot lysis via acoustic cavitation—has emerged as a promising adjuvant therapy. Clinical validation of this ultrasound-mediated strategy comes from a phase II clinical trial, which demonstrated both feasibility and safety when combining microbubble technology with standard intra-arterial thrombolysis (83). Latest research indicates that the stronger cavitation effect induced by dual-frequency ultrasound enhances the removal of retracted clots by up to 85% compared to single-frequency ultrasound, which further demonstrates its potential for treating deep vein thrombosis (DVT) (84).

Beyond conventional thrombolytics such as tissue plasminogen activator (tPA) and pro-urokinase (PUK), the therapeutic potential of plasmin (85) and spermine-NONOate (86) has been evaluated in combination with UTMD. Building on these advancements, current research frontiers in sonothrombolysis focus on engineering multifunctional microbubbles to overcome limitations of traditional thrombolytic agents. Notably, magnetic nanoparticle-conjugated microbubbles represent a significant advancement, enabling spatial guidance via external magnetic fields while maintaining ultrasound-triggered drug release capabilities (87, 88). In parallel, surface functionalization strategies employing fucoidan (89) and arginine-glycine-aspartate-serine (RGDS) peptides enhance thrombus-specific targeting (90). Furthermore, therapeutic gases like nitric oxide (NO) (91, 92) and hydrogen sulfide (H₂S) (93) have been engineered into the gaseous cores of microbubbles, exhibiting dual functionality: thrombus dissolution and mitigation of tissue ischemia-reperfusion injury (IRI). Most recently, a breakthrough approach involving microbubbles integrated with phase-change nanodroplets has emerged. These hybrid systems generate transient micropores within thrombi through acoustic-triggered phase transition, thereby significantly enhancing therapeutic agent penetration (94).

### 3.6 Other applications relevant to CVDs

In China, hypertension stands as the principal modifiable risk factor for CVDs, accounting for approximately 43% of cardiovascular morbidity and mortality (95). Recent advancements in UTMD technology demonstrate promising therapeutic applications for hypertension management. Experimental studies in spontaneously hypertensive rats (SHRs) reveal that UTMD-mediated renal delivery of GRK4-specific siRNA effectively suppresses renal GRK4 expression, thereby restoring dopamine D1 receptor (D1R) signaling pathways. This molecular intervention enhances renal sodium excretion through improved D1R-mediated natriuresis and diuresis, ultimately achieving sustained blood pressure reduction (96).

In the treatment of cerebrovascular diseases, a recent study supports FUS-MB (Focused Ultrasound with Microbubbles) as a minimally invasive therapeutic modality. This technique can therapeutically control the growth and *de novo* formation of cerebral cavernous malformations (CCMs) even without drug delivery (97). Meanwhile, the therapeutic potential of UTMD extends beyond molecular targeting to enhancing cellular therapies. In rodent models of cerebral ischemia induced by middle cerebral artery occlusion (MCAO), UTMD application significantly improves the homing efficiency of intravenously administered bone marrow stromal cells (BMSCs) to ischemic regions. This targeted cellular delivery correlates with reduced infarct volume and improved neurological outcomes. These benefits are potentially mediated through the modulation of matrix metalloproteinase-8 (MMP8) activity—a key regulator of extracellular matrix remodeling in ischemic injury (98).

In an ischemic hindlimb model, low-mechanical-index (MI) microbubble-enhanced ultrasound (MEUS) demonstrated significantly enhanced efficacy in augmenting muscle blood perfusion and reducing necrosis during the early postoperative phase. These effects are primarily attributable to angiogenesis stimulated by low-MI MEUS (99). Additionally, UTMD offers a noninvasive approach to facilitate microRNA delivery, enabling site-specific transfection with minimal systemic or off-target effects. Studies in ischemic hindlimb models show that ultrasound-mediated delivery of miR126-3p–loaded carrier microbubbles to chronically ischemic skeletal muscle promotes enhanced tissue perfusion, increased vascular density, arteriolar formation, and neovessel maturation. Critically, this technique induces no substantial off-target effects in remote organs (100).

## 4 Discussion

Under ultrasound irradiation, commercially available microbubbles exemplified by SonoVue® (Bracco Imaging) demonstrate significant cardioprotective properties even when not loaded with therapeutics. Mechanistic studies attribute these effects to the upregulation of key signaling molecules including vascular endothelial growth factor-α (VEGF-α), insulin-like growth factor-1 (IGF-1), and caveolin-3 (Cav-3), coupled with the liberation of bioactive mediators such as nitric oxide (NO) and adenosine triphosphate (ATP) (104, 106). Notably, UTMD technology has emerged as a promising strategy for enhancing stem cell homing efficacy. Experimental evidence confirms that UTMD not only promotes targeted migration of mesenchymal stem cells (MSCs) to lesion sites but also preserves their fundamental biological characteristics, including proliferation capacity, apoptotic regulation, and cell cycle dynamics (38, 101). Furthermore, microbubble-assisted exosomal delivery systems have demonstrated therapeutic potential in cardiovascular applications. While therapeutic exosome administration shows cardioprotective benefits against toxic injury, limitations persist in cardiac-specific targeting efficiency. UTMD-mediated cavitation effects offer a technological breakthrough by significantly improving myocardial accumulation of exosomal therapeutics, thereby addressing current delivery challenges (62, 63).

Recent breakthroughs in MB nanotechnology have transformed gas-core contrast agents into multifunctional therapeutic systems through advanced engineering strategies. In cardiovascular applications, ligand-functionalized MBs enable molecular-level targeting via specific ligand-receptor interactions. The utilization of dual-modality theranostic contrast agents, compatible with ultrasound and MRI imaging, can be achieved by loading drugs and magnetic materials into the MBs. Furthermore, the polymeric MBs' thicker shell can accommodate a greater quantity of ultrasmall superparamagnetic iron oxide (USPIO) nanoparticles, a property that facilitates the mediation and monitoring of drug delivery (107). It is noteworthy that nanoscale bubbles have the capacity to extend their effects beyond the vascular confinement, thereby facilitating the transport of therapeutic agents across cellular barriers. The utilization of biosynthetic gas vesicles holds considerable promise for facilitating large-scale, non-invasive imaging techniques, thereby enabling the visualization of genetically modified bacteria within living subjects. This development stands to significantly contribute to the advancement of diagnostic and therapeutic cellular agents (108).

Gene delivery systems represent a frontier application with transformative potential in ultrasound-mediated therapy. The process of gene delivery is of paramount importance, as it pertains to the transplantation of foreign DNA to the cells of a host organism. This process is employed within the realm of biomedical research and gene therapy (109). There exist two fundamental gene delivery systems: viral and non-viral. Sonoporation, due to its non-invasiveness, high spatio-temporal resolution and tissue penetration through ultrasound-induced microbubble cavitation, has clear advantages over other modalities. Mechanically, ultrasonic excitation at the appropriate frequency and energy can cause microbubbles to vibrate, expand and collapse, leading to various stable or inertial cavitation effects including microstreams and microjets (110, 111). These physical forces result in the formation of transient and repairable pores in the cellular membrane, thereby facilitating the entry of foreign substances. Pre-clinical investigations have demonstrated that microbubble cavitation-based gene delivery holds considerable promise as a therapeutic modality (112). It has been employed extensively for the delivery of transgenes in the treatment of numerous diseases. Viral vectors are a means of facilitating the transfer of genetic material from one organism to another. This process, known as transduction, involves the use of viruses as vehicles to introduce foreign DNA into the cells of a recipient organism. Despite their capacity to elicit effective gene expression due to their viral configuration, which hinders degradation, numerous studies have demonstrated that the utilization of these carriers is encumbered by several limitations. These limitations encompass immunogenicity (113), off-target delivery (114), and arduous vector production (115). The substantial internal space of ultrasound-responsive microbubbles renders them optimal for use as viral vectors. Consequently, gene transfer strategies that employ acoustically triggered microbubble collapse might effectively circumvent antiviral immune responses while improving targeting. Non-viral gene carriers have gained significant interest due to their comparatively reduced toxicity and immunogenicity in contrast to viral vectors. Nevertheless, the limitations inherent to non-viral carriers include the low levels of protein expression and the inefficient gene transfer efficiency (116). To address this issue, Xie et al. (117) proposed a gene delivery strategy that utilizes ultrasound to directly deliver plasmid DNA into nuclei via gas vesicles (GVs)-based intracellular cavitation. The pDNA-binding GVs are internalized by cells, leading to the formation of intracellular cavitation when exposed to acoustic irradiation, thereby delivering their pDNA payloads into the nuclei.

These innovative targeting approaches - encompassing biochemical specificity, physical guidance, and nanoscale biodistribution - are redefining precision medicine in cardiovascular interventions, particularly for pathologies requiring temporally controlled, site-specific treatment administration. Building upon this paradigm shift in therapeutic delivery, over the past decade, therapeutic research in cardiovascular diseases has predominantly centered on acute myocardial infarction and heart failure, while peripheral arterial disease and cerebrovascular disorders have remained relatively underexplored in preclinical investigations (Table 1). This research gap persists despite growing clinical needs, notably in cerebrovascular therapeutics where conventional drug delivery systems face substantial anatomical barriers. Emerging as a technological breakthrough in this context, the integration of focused ultrasound (FUS) with microbubbles (MBs) has demonstrated significant potential for treating cerebrovascular diseases (118, 119). This noninvasive method enables targeted drug delivery to the brain. At relatively low frequencies, concentrated ultrasound waves can traverse the skull to generate an acoustic field within the brain tissue. When MBs circulate in an ultrasonic field, they undergo oscillation at the same frequency as the ultrasound, a process termed cavitation. In this context, MBs function as cavitation nuclei, moderating the effects of ultrasound while simultaneously inducing transient and reproducible openings of the blood-brain barrier (BBB) (120). The mechanism underpinning FUS-mediated BBB disruption involves the dynamic biomechanical interaction between oscillating MBs and the cerebrovascular structures within the ultrasonic field. This interaction exhibits pronounced parametric sensitivity, being critically dependent on three interrelated factors: 1. acoustic exposure parameters, 2. The physicochemical properties of MBs, and 3. regional vascular density (121). Ultrasound-mediated BBB disruption, supported by well-established mechanisms, preclinical research, and clinical trials, has confirmed its scalability and favorable safety profile. Repeated administrations have not resulted in any clinically detectable tissue damage or neurological complications.

**Table 1:** Preclinical applications of UTMD in Cardiovascular Disease Therapy.

Disease	Study	Shell material	Gas core	Therapeutic molecular	Species	Outcome summary
Myocardial infarction	Deng et al. (2015) (31)	Lipids loaded with ICAM-1 antibody	SF_6_	Ang-1	Rabbit	UTMD-mediated Ang-1 gene delivery improved the efficacy of therapeutic angiogenesis.
	Ghamkhari et al. (2023) (14)	PLGA-HP-PEG-cRGD-platelet	C_6_F_14_	bFGF	Rat	bFGF delivery could notably increase by ultrasound in the MI tissue.
	Su et al. (2015) (39)	Lipids	C_3_F_8_	microRNA-21	Pigs	UTMD-mediated microRNA-21 transfection improved CME-induced cardiac dysfunction.
	Shentu et al. (2018) (30)	Lipids loaded with P-selectin antibody	C_3_F_8_	VEGF	Rat	UTMD-mediated delivery of VEGF_165_ increased myocardial vascular density and improved cardiac function.
	Zhong et al.(2021) (32)	Albumin	C_3_F_8_	miR-150-5p	Rat	OGD-induced primary cardiomyocyte injury was attenuated by UTMD-mediated uptake of miR-150-5p.
	Wang et al.(2023) (35)	Lipids	C_3_F_8_	SERCA2a, Cx43	Rat	A 1:2 ratio of the SERCA2a/Cx43 gene is optimal for keeping the heart's electrophysiological stability.
	Sun et al. (2020) (38)	Lipids	C_3_F_8_	/	Rat	The delivery of PHD2 shRNA-modified BMSCs by UTMD promoted grafted cell activity and increased myocardial angiogenesis.
	Zhang et al. (2017) (33)	Lipids	C_3_F_8_	PHD2	Rat	Ultrasound-mediated gene delivery can enhance shPHD2 gene transfection and improve angiogenesis and contractility.
	Li et al.(2023) (34)	Lipids	C_3_F_8_	Gal-3	Rat	UTMD-mediated Gal-3 shRNA transfection reduced myocardial fibrosis and protected the cardiac ejection function.
	Wang et al.(2022) (36)	Lipids	C_3_F_8_	SERCA2a, Cx43	Rat	UTMD-mediated overexpression of SERCA2a and Cx43 can restore cardiac mechanoelectric function synergistically.
	Sun et al.(2023) (101)	Lipids	C_3_F_8_	/	Rat	UTMD combined with PDGF-BB pretreatment increases the therapeutic effect of grafted BMSCs.
	Yue et al.(2024) (102)	Lipids	C_3_F_8_	KLB	Rat	A UTMD delivery system with CMBs delivering the KLB gene to the heart helps FGF21 alleviate cardiac unfavorable remodeling after AMI.
	Wang et al.(2024) (103)	Biomimetic lipid membrane	liquid fluorocarbon	miRNA-125b	Mouse	MiR-125b modified biomimetic nanoparticles, when combined with UTMD, enhanced cardiac function recovery.
	Cai et al.(2024) (104)	Lipids	SF_6_	/	Rat	UTMD treatment improved left ventricular function in rats with ischemic cardiac dysfunction.
	Yang et al.(2024) (105)	Lipids	C_3_F_8_	*β*-Catenin	Mouse	UTMD-mediated β-catenin gene delivery can reduce the impact of myocardial injury and promote cardiac self-repair after MI.
Ischemia/ reperfusion injury	Mofid et al.(2016) (45)	Lipids	C_3_F_8_	S100A6	Rat	S100A6 overexpression by UTMD resulted in lower mortality and improved left ventricular systolic function.
	Erikson et al.(2017) (43)	Dextrose albumin	PFC	TRAF3IP2	Mouse	Traf3ip2 gene deletion by UTMD can inhibit I/R-induced inflammatory response, myocardial dysfunction, and adverse remodeling.
	Du et al.(2017) (44)	Lipids	C_3_F_8_	GDF11	Mouse	Targeted delivery of GDF11 through UTMD can restore the senescent heart and protect it from ischemic damage.
	Yan et al.(2014) (41)	Lipids loaded with MMP2 antibody	Air	Timp3	Rat	UTMD therapy with this CMB_MMP2_ can improve cardiac repair and ventricular function.
	Kwekkeboom et al.(2016) (42)	Lipids	C_4_F_10_	Antagomir	Mouse	UTMD can increase antagomir inhibitor delivery to cardiomyocytes without causing persistent damage to the heart.
	Dorner et al.(2013) (106)	Lipids	SF_6_	/	Mouse	Ultrasound-mediated stimulation of microbubbles can ameliorate post-infarction remodeling and improve myocardial borderzone vascularization.
	Chen et al.(2016) (46)	Lipids	H_2_S, C_3_F_8_	H_2_S	Rat	Utilizing ultrasound to deliver H_2_S into the myocardium limited the extent of myocardial injury and preserved cardiac function.
Diabetic cardiomyopathy	Zhao et al.(2016) (51)	Lipids	C_3_F_8_	aFGF	Rat	The aFGF-NP + UTMD combined therapy suppressed diastolic dysfunctions, myocardial fibrosis, and metabolic.
	Zhang et al.(2020) (54)	NM-aFGF-PEG- liposomes	C_3_F_8_	aFGF	Rat	NM-aFGF-loaded PEGylated nano-liposomes delivered by UTMD can improve myocardial structural and functional lesions.
	Zhao et al.(2014) (52)	Lipids	C_3_F_8_	aFGF	Rat	bFGF-NP/UTMD combined treatment can improve or even reverse cardiac dysfunction and pathological abnormalities.
	Zhao et al.(2016) (53)	Lipids	C_3_F_8_	bFGF	Rat	The bFGF-loaded liposome combined with UTMD suppressed diastolic dysfunctions, myocardial fibrosis, and metabolic disturbances.
	Guo et al.(2024) (55)	Lipids	C_3_F_8_	SAHH	Rat	Ultrasound-targeted microbubble technology-mediated SAHH gene transfer can prevent diabetes-induced heart dysfunction.
Acute Cardiac Rejection	Yi et al.(2020) (73)	Lipids	C_3_F_8_	Antagomir-155	Mouse	The antagomir-155 delivered by UTMD can lower the levels of cytokines and inflammation and prolong allograft survival time.
	Liu et al.(2019) (71)	Lipids	C_3_F_8_	FK506	Rat	Combining FK506-MBs with UTMD can increase the local drug concentration and enhance rejection efficacy.
	Wang et al.(2020) (72)	Lipids	C_3_F_8_	Galectin-7	Rat	Ultrasound-targeted galectin-7-siRNA knockdown can prevent acute cellular rejection in the early period after allograft heart transplantation.
	Bao et al.(2024) (70)	Lipids	C_3_F_8_	Sirolimus	Rat	Sirolimus-MBs combined with UTMD bolster protection against AR by fostering autophagy, modulating inflammation.
Cardiotoxic damage	Chen et al.(2015) (57)	Lipids	C_3_F_8_	GLP-1	Rat	UTMD-mediated delivery of the GLP-1 gene can reverse established Adriamycin cardiomyopathy by stimulating myocardial regeneration.
	Tian et al.(2017) (58)	Lipids	SF_6_	MaFGF	Rat	Combined application of MaFGF-NP and UTMD prevented myocardial injury induced by DOX and preserved left ventricular systolic function.
	Sun et al.(2020) (63)	Lipids	SF_6_	/	Mouse	UTMD assisted exosomal miR-21 delivery into the heart decreased the cell death induced by DOX, and restored the cardiac function.
	Wang et al.(2023) (65)	Lipids	C_3_F_8_	nuclear receptor ROR*α*	Rat	UTMD-mediated cardiac delivery of RORα optimized protective effects of melatonin on the septic heart.
	Chen et al.(2016) (59)	Lipids	C_3_F_8_	ANGPTL8	Rat	UTMD-mediated delivery of ANGPTL8 reversed established ADM cardiomyopathy.
	Lee et al.(2014) (60)	Lipids	C_3_F_8_	survivin	Rat	Survivin gene therapy via UTMD can attenuate the progression of LV systolic dysfunction in DOX cardiomyopathy.
	Zhou et al.(2021) (61)	Lipids	SF_6_	aFGF	Rat	The aFGF-NP + CPMBs combined with UTMD could effectively antagonize cardiac damage induced by DOX.
	Chen et al.(2024) (62)	Lipids	SF_6_	/	Mouse	UTMD-assisted exosomal delivery of siHomox1 significantly reduced ferroptosis and cardiotoxicity caused by doxorubicin.
Cardiac hypertrophy	Qin et al.(2023) (24)	Lipids	C_3_F_8_	Sirt3	pig	UTMD-mediated targeted delivery of Sirt3 can repress cardiac hypertrophy, and preserve cardiac function.
	Kopechek et al.(2019) (67)	Lipids	C_4_F_10_	antimiR-23a	Mouse	UTMC could target the delivery of antimiR-23a to cardiomyocytes, suppress cardiac hypertrophy, and preserve cardiac function.
Atherosclerotic plaque	Wu et al. (2023) (77)	Lipids	C_3_F_8_	microRNA-145	Mouse	The treatment with miR-145 via UTMD reduced atherosclerotic plaque formation.
	Zhou et al. (2022) (76)	Lipids	SF_6_	Rapamycin	Mouse	RAP @ PLT nanoparticles combined with SnonVue can improve plaque stability and inhibit atherosclerotic plaques.
	Yang et al. (2020) (79)	Lipids	SF_6_	GSK-3β	Rabbit	Downregulation of GSK-3β expression by UTMD suppressed vulnerable plaque factors and inflammation.
	Su et al. (2017) (81)	/	/	TGF-β1 and TIMP1	Rabbit	UTMD combined with dual targeting of TGF-β1 and TIMP1 recombinant adeno-associated virus can stabilize atherosclerotic vulnerable plaques.
	Yang et al. (2019) (80)	Lipids	C_3_F_8_	IL-8	Rabbit	The treatment with IL-8 monoclonal antibody via UTMD inhibited the inflammatory response and increased plaque stability.
	Yuan et al. (2018) (78)	Lipids loaded with ICAM-1 antibody	PFC	Endostar	Mouse	ICAM1-targeted and Endostar-loaded microbubbles with UTMD reduced atherosclerotic plaque area.
	Hu et al. (2023) (75)	Platelet membrane-coated siNox2-lipids	C_5_F_12_	Nox2	Mouse	The platelet membrane-coated nanobubbles loaded with small siNox2 can efficiently slow the progression of plaques.
Hypertension	Huang et al. (2016) (96)	Lipids	C_3_F_8_	GRK4	Rat	UTMD-mediated renal GRK4 siRNA delivery can reduce GRK4 expression and lower BP in spontaneously hypertensive rats.
Thrombosis	Wang et al.(2020) (88)	SiO2-tPA, Fe_3_O_4_	Air	tPA	Mouse	The magnetic nanoparticle-shelled microbubble not only improved the therapeutic efficacy but also accelerated the lytic rate.
	Zhong et al.(2021) (93)	Lipids	H_2_S, C_3_F_8_	H_2_S	Rat	H_2_S-loaded microbubbles combined with ultrasound can dissolve thrombi and protect against skeletal muscle IRI.
	Pan et al.(2022) (94)	Nanodroplets- coated lipids	SF_6_	PUK	Mouse	Nanodroplet-coated microbubbles showed high diffusion and thrombolysis efficiency.
	Zhang et al.(2021) (87)	SDS-Fe_3_O_4_ nanoparticles	Air	/	/	Combining nanodroplets with MMBs significantly enhanced the *in vitro* lysis of both unretracted clots and retracted clots in a flow model.
	Fournier et al.(2023) (89)	Fucoidan-loaded PIBCA	C_4_F_10_	tPA	Mouse	The rtPA-loaded fucoidan MBs to be over 50% more efficient than regular free tPA injection for stroke resolution.
	Corro et al.(2022) (86)	Lipids	C_3_F_8_	Spermine NONOate	Rat	The spermine-NONOate-loaded microbubbles achieved large-vessel thrombolysis and protection against tissue ischemia.
	Kandadai et al.(2014) (85)	Plasmin-loaded lipids	Air	Plasmin	/	With US exposure, the average clot breaking down with PELIP was 31% higher than without US exposure and 15% higher than with tPA treatment.
	Zheng et al.(2022) (90)	RGDS-load lipids	SF_6_	PUK	Sheep	The interventional sonothrombolysis shown to be more efficient and safer than other thrombolysis procedures for LVAD.
	Liang et al.(2022) (91)	Lipids	SF_6_, NO	NO	Rat	NO-loaded microbubbles achieved large vessel thrombolysis and protection against IRI.
	Shi et al.(2024) (92)	Lipids	SF_6_, NO	NO	Rat	With real-time tracking of contrast-enhanced ultrasound, NO-MBs UTMD therapy can restore blood perfusion in the embolized tissue in time.
Acute cerebral infarction	Bai et al.(2024) (98)	Lipids	SF_6_	/	Rat	UTMD facilitates the migration and homing of BMSC into the brain, improves therapeutic outcomes in an ACI rat model.
Cerebral cavernous malformations	Fisher et al.(2025) (97)	Albumin	C_3_F_8_	/	Mouse	FUS-MB safely arrests murine CCM growth and prevents *de novo* CCM formation.
Hindlimb ischemia	Cao et al.(2024) (100)	Lipids	C_4_F_10_	miR-126-3p	Rat	Gene delivery of miR126-3p–bearing microbubbles improves tissue perfusion and vascular density in chronically ischemic skeletal muscle.
	Zhu et al.(2024) (99)	Lipids	C_3_F_8_	/	Mouse	Low MI MEUS significantly enhanced early post-HLI muscle perfusion and reduced necrosis, primarily via angiogenesis.

C_3_F_8_, perfluoropropane; SF_6_, sulfur hexafluoride; C_4_F_10_, perfluorobutane; NO, nitrogen monoxide; H_2_S, hydrogen sulfide; C_6_F_14_, perfluorohexane; C_5_F_12_, perfluoropentane; VSMCs, murine ascular smooth muscle cells; RAP, rapamycin; RLT, platelets; (TGF)-β1, transforming growth factor; TIMP1, tissue inhibitors of metalloproteinase; ICAM-1, intercellular adhesion molecule 1; Ang-1, angiopoietin-1; bFGF, basic fibroblast growth factor; HUVECs, human umbilical vein endothelial cells; CME, coronary microembolization; PDCD4, programmed cell death 4; TNF-α, tumor necrosis factor α; NF-κB, nuclear factor kappa-B; VEGF, vascular endothelial growth factor; OGD, Oxygen–glucose deprivation; TTC5, tetratrico peptide repeat domain 5; BMSC, bone marrow stem cell; PHD2, prolyl hydroxylase domain protein 2; CMBs, cationic microbubbles; Gal-3, Galectin-3; aFGF, acidic fibroblast growth factor; NP, nanoparticles; NM, non-mitogenic; MB, microbubbles; US, ultrasound; CMECs, cardiac microvascular endothelial cells; MaFGF, non-mitogenic acidic fibroblast growth factor; DOX-CM, doxorubicin-induced cardiomyopathy; ADM, Adriamycin; PLGA, poly(lactic-co-glycolic acid); HP, heparin; PEG, polyethylene glycol; cRGD, cyclic arginine-glycine-aspartate; RAP, rapamycin; PLGA, lactic-co-glycolic acid; PLT, platelet; CPMBs, cationic lipid microbubbles; LV, left ventricular; UTMC, ultrasound-targeted microbubble cavitation; PFC, perfluorocarbon; tPA, tissue plasminogen activator; SiO2-tPA, tPA-containing mesoporous silica nanoparticles; IRI, ischemia/reperfusion injury; PUK, recombinant human urokinase pro; NSt, rtPA-functionalized asymmetrical nanostars; MMBs, magnetic microbubbles; PIBCA, Poly-isobutyl cyanoacrylate; PELIP, plasmin-loaded echogenic liposomes; DDFP, dodecafluoropentane; LVAD, left ventricular assist device; PUK, pro-urokinase; RGDS, arginine-glycin-aspartate-serine; PDGF, platelet-derived growth factor. ACI, acute cerebral infarction; KLB, β-klotho; AMI, acute myocardial infarction; SAHH, S-Adenosylhomocysteine hydrolase; MI, mechanical index; MEUS, microbubble enhanced ultrasound; HLI, hind limb ischemia; FUS-MB, focused ultrasound-microbubble; CCM, cerebral cavernous malformations.

Beyond current clinical trials of focused ultrasound-mediated blood-brain barrier opening for brain tumors, Alzheimer's disease, Parkinson's disease, and amyotrophic lateral sclerosis, emerging applications target neuropsychiatric disorders (including major depression and substance addiction) and central pain syndromes. To translate these diverse applications into clinical practice, integrated systems enabling real-time therapy control and safety validation become paramount. The combination of clinically approved MRI-guided focused ultrasound (MRgFUS) with engineered, shell-optimized microbubbles establishes a robust platform for evaluating therapeutic interventions across diverse patient groups. Real-time MRI monitoring, coupled with advanced microbubble formulations, further provides critical evidence regarding safety and feasibility, particularly for cohorts with neurological impairments that require therapies targeting the central nervous system (CNS) (122).

UTMD embodies precision medicine principles through its unique mechanism, demonstrating substantial clinical promise. Preclinical studies validate its safety profile, with microbubbles exhibiting high circulatory stability and biocompatibility *in vivo*. Surface modification strategies further enhance microbubble localization, enabling UTMD to achieve tissue-specific targeting. Nevertheless, technical challenges persist regarding clinical translation.

The current absence of mass-production manufacturing methodologies for ultrasound-responsive microbubbles remains a critical barrier. Furthermore, refining the physicochemical composition of these contrast agents represents a pivotal challenge for their clinical translation. Specifically, next-generation formulations must simultaneously demonstrate enhanced drug-loading efficiency, consistent acoustic responsiveness, and convenient production-preservation-transportation workflows to meet clinical demands. Beyond these engineering bottlenecks, the transition from preclinical validation to human applications introduces additional biological complexities. While the feasibility of UTMD has been extensively validated in rodent models, the inherent anatomical complexity and physiological heterogeneity of human systems necessitate a phased translational approach. This progression should prioritize rigorous validation in large-animal models before advancing to controlled human trials, ensuring interspecies compatibility in microbubble-mediated therapeutic delivery.

The clinical implementation of UTMD necessitates stringent control over interdependent acoustic parameters - including frequency, mechanical index (MI), pulse duration, and duty cycle (5) - which require systematic calibration to achieve therapeutic bioeffects (mechanical/thermal) while avoiding off-target tissue damage. This precision engineering challenge is particularly evident in focused ultrasound-mediated BBB opening, where safety profiles exhibit significant context-dependency influenced not only by acoustic variables but also by microbubble characteristics (size distribution, shell composition) and vascular anatomical constraints (123). The complex interplay between inertial cavitation thresholds, microbubble oscillation dynamics, and non-linear acoustic interactions mandates the development of multiparametric optimization frameworks. Such protocols must balance BBB permeability enhancement with preservation of neurovascular integrity, requiring real-time feedback systems capable of adjusting sonication parameters in response to dynamic physiological feedback. Emerging evidence indicates that FUS-mediated BBB opening may inadvertently provoke neuroinflammatory cascades (124). Therefore, a critical technological barrier persists: the absence of robust, non-invasive tools for real-time tracking of neuroinflammatory dynamics *in vivo*. Addressing this unmet need requires prioritized development of targeted molecular imaging probes capable of selectively detecting inflammation-associated biomarkers. Such innovations would not only enable safety profiling during FUS interventions but also facilitate precision modulation of treatment parameters to optimize therapeutic outcomes while mitigating adverse effects.

UTMD has demonstrated unique advantages in cardiovascular disease management, particularly through its capacity for targeted drug/gene delivery mediated by ultrasound-triggered microbubble cavitation. The convergence of these platforms could address critical challenges in cardiovascular therapies, including tissue specificity, delivery efficiency, and safety. However, clinical translation requires systematic validation of their synergistic mechanisms, long-term biocompatibility, and spatiotemporal control precision. By advancing these ultrasound-mediated technologies, we may pioneer a new era in cardiovascular care—from molecular level interventions to organ monitoring—driving a shift towards personalised medicine.
